# Body size and thyroid cancer in two million Norwegian men and women

**DOI:** 10.1038/sj.bjc.6603249

**Published:** 2006-07-11

**Authors:** A Engeland, S Tretli, L A Akslen, T Bjørge

**Affiliations:** 1 Division of Epidemiology, Norwegian Institute of Public Health, PO Box 4404, N-0403 Oslo, Norway; 2 Department of Public Health and Primary Health Care, Section for Epidemiology and Medical Statistics, University of Bergen, N-5018 Bergen, Norway; 3 The Cancer Registry of Norway, Institute of Population-Based Cancer Research, Montebello, N-0310 Oslo, Norway; 4 The Gade Institute, Section for Pathology, University of Bergen, N-5020 Bergen, Norway; 5 Division of Epidemiology, Norwegian Institute of Public Health, N-5018 Bergen, Norway

**Keywords:** thyroid cancer, body mass index, height, cohort study, Norway

## Abstract

We investigated relations between measured body mass index (BMI) and stature and thyroid cancer (3046 cases) in a large Norwegian cohort of more than two million individuals. The risk of thyroid cancer, especially of the papillary and follicular types, increased moderately with increasing BMI and height in both sexes.

Several types of malignancies have been associated with increased weight, as summarised recently (IARC Working Group on the Evaluation of Cancer-Preventive Strategies, 2002). As thyroid cancer is relatively rare, with only a few studies of its association with obesity, the working group did not include it in its review.

In 1993–1997, thyroid cancer comprised 0.5 and 1.4% of all male and female cancer cases, respectively, in the Nordic countries (Møller *et al*, 2002). In Norway, the (world) age-adjusted incidence rates in 2004 were 1.5 and 5.1 per 100 000 persons per year in men and women, respectively (The Cancer Registry of Norway, 2006), the higher rates among women being typical (Parkin *et al*, 2005). In the US, the rates of thyroid cancer are significantly higher and increasing. In 2001, the age-adjusted incidence rates were 4.2 and 12 per 100 000 persons per year in males and females (National Cancer Institute, 2005).

In the present study, we used measurements from Norwegian health surveys to examine the relations between body mass index (BMI) and stature and the risk of thyroid cancer. We have recently published similar analyses for a range of cancers (Engeland *et al*, 2003a, 2003b and 2003c, 2004, 2005; Bjørge *et al*, 2004).

## MATERIALS AND METHODS

The study population and the methods have been described elsewhere (Bjørge *et al*, 2004). In a number of Norwegian health surveys during 1963–2001, height and weight were measured in 2 001 727 persons (963 709 men and 1 038 018 women) aged 20–74 years in a standardised way by a trained staff. A major part of the health surveys in the period 1963–1975 was included in a nationwide tuberculosis screening programme (Waaler, 1984), which was compulsory for all Norwegians aged 15 years and above; attendance was about 85%. In 1963–1964 and in 1972–2001, height and weight were also measured in other health surveys connected with coronary heart disease in different parts of Norway (Bjartveit *et al*, 1979; Bjartveit, 1997). The attendance in these surveys in the mid-1970s was 85–90% and about 75% in the mid-1990s (Bjartveit, 1997).

Deaths, emigrations and cases of thyroid cancer (International Classification of Diseases, seventh revision (ICD-7): 194) in this cohort were identified by linkage to the Death Registry at Statistics Norway (Statistics Norway, 2006) and to the Cancer Registry of Norway (The Cancer Registry of Norway, 2006). Both these registries are population based and cover the entire Norwegian population. A unique 11-digit identification number assigned to all individuals living in Norway after 1960 facilitated the linkages.

The present study included only histologically verified thyroid cancers. Persons with a diagnosis of thyroid cancer before the height and weight measurements were excluded (*n*=709). In the analyses, the persons in the cohort were followed from the date of measurement until the date of cancer diagnosis, emigration, age 100 years, death or until 31 December 2003. Altogether, 2 001 018 persons were eligible for the study. A small number of these (39 men and 32 women) were lost to follow-up.

### Statistical methods

Cox's proportional hazards regression models (Cox and Oakes, 1984), with time since measurement as the time variable, were fitted to obtain relative risk estimates of cancer. In the analyses, categorised variables for age at measurement, year of birth, BMI ((weight in kilograms)/(height in metres)^2^) and height were included. Body mass index was categorised using the WHO categorisation (World Health Organization Consultation on Obesity, 1998): BMI <18.5 (underweight), 18.5–24.9 (normal), 25.0–29.9 (preobese/overweight) and ≥30.0 kg/m^2^ (obese).

Analyses were also performed treating BMI and height, respectively, as continuous variables to test for trend in thyroid cancer risk. Separate analyses were performed for papillary, follicular, medullary, anaplastic, other carcinomas and other malignant tumours. All these analyses were performed with the statistical program package SPSS (SPSS Inc., SPSS for Windows, Release 12.0.2.2004). The results were presented as relative risks (RR) of cancer with 95% confidence intervals (CI). The hazard functions of thyroid cancer by BMI and height in the Cox model were estimated using spline functions in S-plus (Insightful Corporation, 2002), with 4 d.f.

## RESULTS

The 2 000 947 persons (963 523 men and 1 037 424 women) included in this study were followed for on average 23 years (maximum 41 years), constituting 47 million person-years (Table 1). The mean age at measurement was 44 years, and the proportions of obesity were 6 and 13% in men and women, respectively. During follow-up, 3046 thyroid cancer cases were diagnosed among the study subjects on average 15 years after the measurements. The mean age at diagnosis was 62.2 and 57.7 years in men and women, respectively.

The risk of thyroid cancer increased moderately by increasing BMI in both sexes (Table 2). The RR of thyroid cancer per unit increase in BMI was 1.03 (95% CI: 1.00–1.05) in men and 1.02 (95% CI: 1.01–1.03) in women. Excluding the first 5 years of follow-up did not change these results. Splitting the upper BMI category in women gave RRs of 1.27 (95% CI: 1.11–1.47), 1.33 (95% CI: 1.03–1.73) and 1.38 (95% CI: 0.83–2.30) with BMI of 30.0–34.9, 35.0–39.9 and 40 or above, respectively, compared with normal-weighted women.

Histology-specific analyses revealed that the relative risk of follicular carcinoma increased more than the risk of papillary carcinoma with increasing BMI. Further, the risk of medullary carcinoma tended to decrease with increasing BMI in both sexes, being significant among females only. The RR of medullary carcinoma per unit increase in BMI was 0.94 (95% CI: 0.85–1.04) in men and 0.91 (95% CI: 0.86–0.97) in women. The number of cases was low (52 and 107 medullary carcinomas in men and women, respectively). The anaplastic carcinomas showed a strong positive association with BMI in men.

The risk of thyroid cancer increased with increasing height in both sexes (Table 2). The RR of thyroid cancer associated with 10 cm increase in height was 1.18 (95% CI: 1.05–1.32) in men and 1.22 (95% CI: 1.13–1.31) in women.

Among males, the elevated risk associated with increased BMI was confined to those measured at the age of 50–74 years (Table 3).

**Figure 1:**
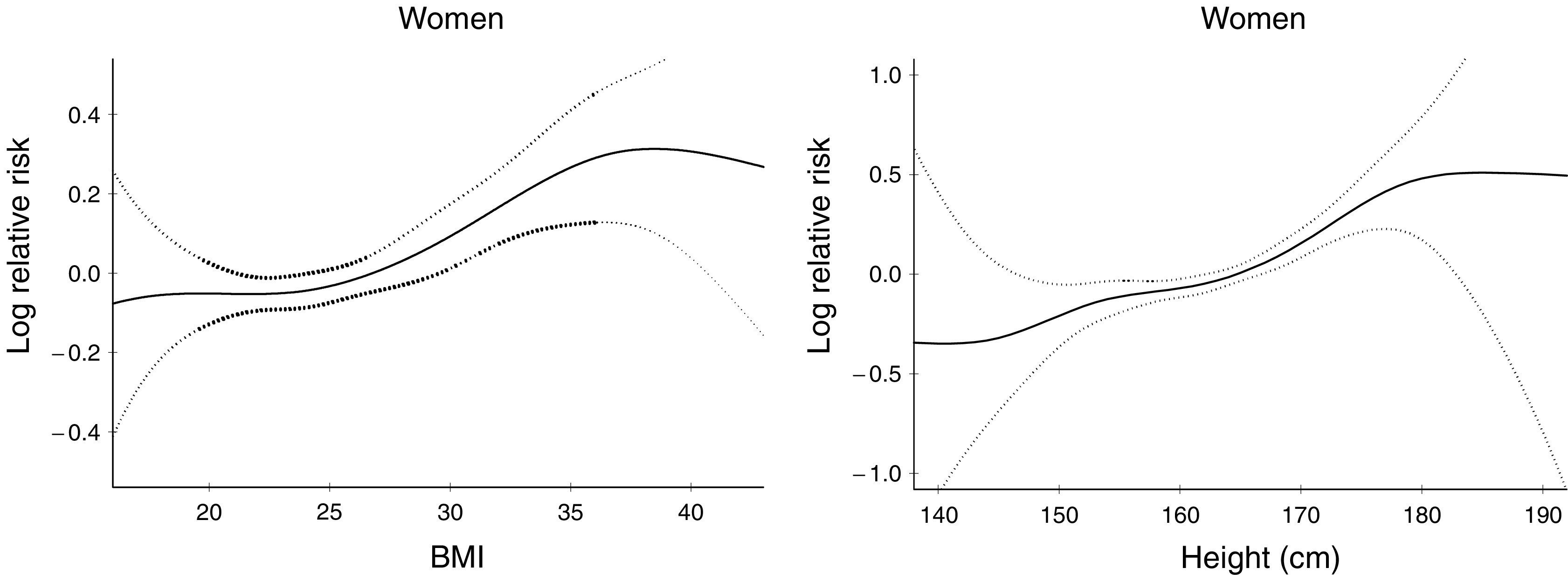
Logarithm of the relative risk of thyroid cancer, with 95% CIs, from penalised spline functions with 4 d.f. Adjusted for birth cohort and age at measurements.

The associations between BMI and stature and the risk of thyroid cancer in women are illustrated in Figure 1. The risk of thyroid cancer increased with increasing height and BMI above 25kg/m^2^.

## DISCUSSION

In this large cohort, including more than two million individuals, we explored the associations between BMI and height and the occurrence of thyroid cancer. The risk was found to be moderately elevated with increasing BMI and height in both sexes among the follicle-cell derived (papillary and follicular) carcinomas. In medullary carcinomas, an opposite trend was present for BMI, pointing to a different pathogenetic influence.

The associations between BMI and height with thyroid cancer have been difficult to explore in previous small studies owing to the low incidence of this disease. In addition to the large size of the present cohort, we also had an almost complete follow-up with regard to thyroid cancer incidence and deaths. This was made possible by linkages to population-based registries of high quality. Only 0.04% of the study cohort (71 persons) was lost to follow-up, and 0.6% was censored by emigration from Norway.

After a pooled analysis of 12 case–control studies, including 2473 cases, Dal Maso *et al* (2000) previously found a moderately increased risk of thyroid cancer related to height and weight at diagnosis. Dal Maso *et al* (2000) used self-reported values for height and weight during the late teens and at diagnosis. In the present study, we relied on height and weight measurements performed on average 15 years before the diagnosis of thyroid cancer. Hence, our estimates were not diluted by a possible influence of individuals weight on the disease.

Iribarren *et al* (2001) evaluated several potential predictors of thyroid cancer in a large American cohort with long follow-up, including more than 200 000 persons. Despite the large size of the cohort and the long follow-up, only 196 thyroid cancers were observed. In this study, no association between BMI, height or weight gain and thyroid cancer was observed.

Dal Maso *et al* (2000) hypothesised that an association between BMI and thyroid cancer could be owing to a relationship with steroid hormones or other endocrine factors. However, they observed an association between BMI and thyroid cancer of similar magnitude in older and younger women. Also in the present study, there was no difference in the association between BMI and thyroid cancer in older and younger women.

Like Dal Maso *et al* (2000), we observed a moderately increasing risk of thyroid cancer with increasing height. This association may be owing to dietary influence during childhood or adolescence and possible interactions with growth factors.

In conclusion, the present study showed that the risk of thyroid cancer increased moderately with increasing BMI and height in both males and females.

## Figures and Tables

**Table 1:** Number of observed cases of thyroid cancer, person-years and overall incidence rates

	**Men**			**Women**		
**Variable**	**No. of cases**	**Person-years**	**Incidence rate^a^**	**No. of cases**	**Person-years**	**Incidence rate^a^**
*Time since measurement*						
0–4	111	4 703 991	2	373	5 101 421	7
5–9	129	4 185 673	3	408	4 626 652	9
10–14	138	3 538 321	4	388	4 037 840	10
15–19	117	2 973 578	4	380	3 549 515	11
20–24	115	2 513 972	5	302	3 129 190	10
25–29	110	2 099 195	5	254	2 710 836	9
≥30	58	1 537 708	4	163	2 154 886	8
						
*Age at measurement*						
20–29	102	4 857 330	2	424	5 592 525	8
30–39	125	4 646 552	3	445	5 274 591	8
40–49	249	6 527 602	4	585	7 032 741	8
50–59	181	3 457 135	5	471	4 461 529	11
60–69	103	1 702 823	6	279	2 421 213	12
70–74	18	360 996	5	64	527 742	12
						
*Year of birth*						
<1900	23	468 282	5	71	685 680	10
1900–1909	103	1 775 347	6	290	2 553 276	11
1910–1919	191	3 513 545	5	478	4 548 443	11
1920–1929	200	4 993 652	4	491	5 567 701	9
1930–1939	119	4 316 383	3	379	4 830 007	8
1940–1949	108	4 645 330	2	411	5 106 836	8
≥1950	34	1 839 899	2	148	2 018 398	7
						
*BMI (kg/m^2^)*						
<18.5	2	138 637	1	30	549 113	5
18.5–24.9	412	12 572 703	3	1187	14 418 940	8
25.0–29.9	322	7 837 913	4	710	7 426 823	10
≥30.0	42	1 003 185	4	341	2 915 463	12
						
*Height (cm)* b						
<150				28	366 766	8
<160/150–159	8	143 556	6	628	7 042 763	9
160–169	116	3 323 324	3	1,247	14 591 487	9
170–179/≥170	429	11 760 603	4	365	3 309 325	11
≥180	225	6 324 955	4			
						
Total	778	21 552 438	4	2268	25 310 340	9

Abbreviations: BMI=body mass index.

a Number of thyroid cancer cases per 100 000 person-years.

b Split categories pertain to men and women, respectively.

**Table 2:** RR of thyroid cancer with 95% CI obtained in Cox's regression analyses; age at measurements and birth cohort were included in the model in addition to either BMI or height^a^

		**Papillary carcinoma**		**Follicular carcinoma**		**Medullary carcinoma**		**Anaplastic carcinoma**		**All thyroid cancers**	
**Sex**	**Variable**	**RR**	**95% CI**	**RR**	**95% CI**	**RR**	**95% CI**	**RR**	**95% CI**	**RR**	**95% CI**
Men		479 cases		111 cases		52 cases		54 cases		778 cases	
	*BMI (kg/m^2^)*										
	<18.5	0.36	0.05–2.60	0.00	0.00-∞	0.00	0.00-∞	0.00	0.00-∞	0.47	0.12–1.87
	18.5–24.9	1.00	Referent	1.00	Referent	1.00	Referent	1.00	Referent	1.00	Referent
	25.0–29.9	1.14	0.94–1.38	1.08	0.73–1.61	0.81	0.45–1.45	1.53	0.87–2.69	1.12	0.97–1.30
	≥30.0	1.11	0.73–1.69	1.66	0.82–3.37	0.69	0.16–2.91	2.33	0.88–6.20	1.14	0.82–1.56
	*Test for trend*^b^ Height (cm)	*P*=0.05		*P*=0.05		*P*=0.3		*P*=0.03		*P*=0.03	
	<160	1.50	0.61–3.64	0.00	0.00-∞	2.45	0.33–18	1.88	0.25–14	1.24	0.61–2.49
	160–169	0.82	0.62–1.08	0.68	0.40–1.16	1.01	0.47–2.17	0.95	0.47–1.90	0.81	0.66–1.00
	170–179	1.00	Referent	1.00	Referent	1.00	Referent	1.00	Referent	1.00	Referent
	≥180	1.29	1.06–1.59	1.02	0.65–1.61	1.22	0.64–2.32	0.78	0.38–1.61	1.17	0.99–1.38
	Test for trend^b^	*P*=0.001		*P*=0.5		*P*=0.5		*P*=0.4		*P*=0.005	
Women		1456 cases		421 cases		84 cases		107 cases		2268 cases	
	*BMI (kg/m^2^)*										
	<18.5	0.59	0.38–0.94	1.02	0.48–2.17	0.00	0.00-∞	3.02	0.93–9.80	0.68	0.47–0.98
	18.5–24.9	1.00	Referent	1.00	Referent	1.00	Referent	1.00	Referent	1.00	Referent
	25.0–29.9	1.12	0.99–1.27	1.13	0.90–1.42	0.43	0.25–0.74	1.24	0.79–1.95	1.08	0.98–1.20
	≥30.0	1.19	1.01–1.41	1.63	1.24–2.15	0.35	0.16–0.79	1.65	0.97–2.80	1.29	1.13–1.46
	*Test for trend*^b^ *Height (cm)*	*P*=0.008		*P*=0.002		*P*=0.004		*P*=0.3		*P*<0.001	
	<150	0.76	0.45–1.27	1.09	0.54–2.23	0.00	0.00-∞	0.30	0.04–2.16	0.76	0.52–1.11
	150–159	0.92	0.81–1.05	1.07	0.86–1.34	0.98	0.59–1.63	0.56	0.36–0.89	0.95	0.86–1.05
	160–169	1.00	Referent	1.00	Referent	1.00	Referent	1.00	Referent	1.00	Referent
	≥170	1.37	1.19–1.58	1.34	0.99–1.80	2.78	1.57–4.91	1.33	0.71–2.49	1.40	1.24–1.58
	Test for trend^b^	*P*<0.001		*P*=0.2		*P*=0.01		*P*=0.001		*P*<0.001	

Abbreviations:BMI=body mass index, CI=confidence interval, RR=relative risk.

a Year of birth and age at measurement were included as continuous variables when number of cases was less than 150.

b BMI and height, respectively, were included as continuous variables.

**Table 3:** RR of thyroid cancer with 95% CI obtained in Cox's regression analyses; age at measurements and birth cohort were included in the model in addition to either body mass index or height. Analysis stratified on age at measurement

	**Thyroid cancer**			
	**Aged 20–49 years**		**Aged 50–74 years**	
	**RR**	**95% CI**	**RR**	**95% CI**
*Men*	476 cases		302 cases	
BMI (kg/m^2^)				
<18.5	0.37	0.05–2.67	0.61	0.09–4.36
18.5–24.9	1.00	Referent	1.00	Referent
25.0–29.9	1.08	0.89–1.30	1.20	0.95–1.52
≥30.0	0.99	0.61–1.60	1.29	0.84–2.00
Test for trend^a^	*P*=0.2		*P*=0.09	
Height (cm)				
<160	1.05	0.26–4.21	1.29	0.57–2.92
160–169	0.88	0.65–1.20	0.76	0.58–1.01
170–179	1.00	Referent	1.00	Referent
≥180	1.17	0.96–1.42	1.19	0.87–1.62
Test for trend^a^	*P*=0.06		*P*=0.03	
		
*Women*	1,454 cases		814 cases	
BMI (kg/m^2^)				
<18.5	0.63	0.42–0.94	1.03	0.46–2.31
18.5–24.9	1.00	Referent	1.00	Referent
25.0–29.9	1.10	0.97–1.25	1.07	0.91–1.25
≥30.0	1.25	1.04–1.51	1.31	1.09–1.57
Test for trend^a^	*P*=0.04		*P*=0.001	
Height (cm)				
<150	0.95	0.51–1.77	0.70	0.44–1.13
150–159	0.91	0.79–1.04	1.00	0.87–1.16
160–169	1.00	Referent	1.00	Referent
≥170	1.34	1.18–1.53	1.67	1.26–2.21
Test for trend^a^	*P*<0.001		*P*=0.002	

Abbreviations:BMI=body mass index, CI=confidence interval, RR=relative risk.

a BMI and height, respectively, were included as continuous variables.
